# PNNGS, a multi-convolutional parallel neural network for genomic selection

**DOI:** 10.3389/fpls.2024.1410596

**Published:** 2024-09-03

**Authors:** Zhengchao Xie, Lin Weng, Jingjing He, Xianzhong Feng, Xiaogang Xu, Yinxing Ma, Panpan Bai, Qihui Kong

**Affiliations:** ^1^ Research Center for Life Sciences Computing, Zhejiang Laboratory, Hangzhou, China; ^2^ Key Laboratory of Soybean Molecular Design Breeding, Northeast Institute of Geography and Agroecology, Chinese Academy of Sciences, Changchun, China; ^3^ School of Computer Science and Technology, Zhejiang Gongshang University, Hangzhou, China

**Keywords:** deep learning, parallelism, genomic selection, plant breeding, stratified sampling

## Abstract

Genomic selection (GS) can accomplish breeding faster than phenotypic selection. Improving prediction accuracy is the key to promoting GS. To improve the GS prediction accuracy and stability, we introduce parallel convolution to deep learning for GS and call it a parallel neural network for genomic selection (PNNGS). In PNNGS, information passes through convolutions of different kernel sizes in parallel. The convolutions in each branch are connected with residuals. Four different L*p* loss functions train PNNGS. Through experiments, the optimal number of parallel paths for rice, sunflower, wheat, and maize is found to be 4, 6, 4, and 3, respectively. Phenotype prediction is performed on 24 cases through ridge-regression best linear unbiased prediction (RRBLUP), random forests (RF), support vector regression (SVR), deep neural network genomic prediction (DNNGP), and PNNGS. Serial DNNGP and parallel PNNGS outperform the other three algorithms. On average, PNNGS prediction accuracy is 0.031 larger than DNNGP prediction accuracy, indicating that parallelism can improve the GS model. Plants are divided into clusters through principal component analysis (PCA) and K-means clustering algorithms. The sample sizes of different clusters vary greatly, indicating that this is unbalanced data. Through stratified sampling, the prediction stability and accuracy of PNNGS are improved. When the training samples are reduced in small clusters, the prediction accuracy of PNNGS decreases significantly. Increasing the sample size of small clusters is critical to improving the prediction accuracy of GS.

## 1 Introduction

In recent years, the yield growth rate of rice [*Oryza sativa* L.] and maize [*Zea mays* L.] has gradually slowed (Yu et al., 2022; Tian et al., 2021). Phenotypic selection (PS) identifies the best individuals based on phenotypic values estimated from performance in evaluation trials. It requires a long period and may take many years to obtain plants with the desired resistance (Bandillo et al., 2023). In tea variety breeding, PS takes more than 16 years. A tea breeding program to meet commercial requirements could take more than 40 years (Lubanga et al., 2023). Genomic selection (GS) is a breeding method based on high-density molecular markers (McGowan et al., 2021). GS estimates individual breeding values through phenotypes and single nucleotide polymorphisms (SNPs). Seedlings are selected based on their breeding value to shorten the generation interval and speed up the breeding process (Cappetta et al., 2020). GS improves the breeding selection accuracy and saves costs (Beyene et al., 2021). GS has accurate prediction results for complex traits with low heritability (Bhat et al., 2016; Merrick and Carter, 2021). Genome technology has also been implemented to guide breeding practices (Jannink et al., 2010). GS provides new opportunities for establishing wheat [*Triticum aestivum* L.] hybrid breeding programs (Zhao et al., 2015). In GS breeding, it is necessary to construct a training population (TP) (Somo et al., 2020). We obtain high-quality phenotypes through precise measurements. A genotype-to-phenotype prediction model is established based on the TP’s phenotype and genotype (Karlsen et al., 2023; van Hilten et al., 2021; Danilevicz et al., 2022). Finally, the genomic estimated breeding value (GEBV) of the predictive group (PG) is calculated through the statistical model (Melnikova et al., 2021). Each PG is evaluated and utilized according to its GEBV (Park et al., 2020). GS has a selective advantage over PS in soybean yield. However, GS reduces genetic diversity (Bandillo et al., 2023).

An early algorithm applied to GS was the best linear-unbiased prediction (BLUP). Subsequently, various algorithms were developed based on BLUP. Genomic best linear-unbiased prediction (GBLUP) assumes that all marker effects have equal variance (Ren et al., 2021). The ridge-regression best linear unbiased prediction (RRBLUP) GS model combines all marker information to predict GEBVs while implementing a penalty function to limit the additive contribution of each marker. Penalties apply equally to all markers for small and large effect genomic components (Rice and Lipka, 2019). When we assume the variance of the marker effect is some prior distribution, the model becomes a Bayesian approach. Currently, Bayesian methods have been developed into Bayesian A, Bayesian B, Bayesian Cπ, Bayesian LASSO, and Bayesian ridge regression (Desta and Ortiz, 2014). Bayesian B outperforms GBLUP as the number of quantitative trait loci decreases (Daetwyler et al., 2010). BLUP mainly considers the additive effects of multiple genes, and does not consider dominant effects and interaction effects. For complex agronomic traits, the BLUP prediction accuracy is less than 0.5.

To further improve the accuracy of phenotype prediction, machine learning is introduced into GS. Machine learning is a data learning algorithm that does not rely on rule design. It processes large amounts of historical data and autonomously identifies patterns in the data. In a comparative study, the phenotypic prediction accuracy of random forest (RF), stochastic gradient boosting (SGB), and support vector machines (SVMs) were all around 0.5 (Ogutu et al., 2011). Machine learning algorithms are generally more complex than linear algorithms. However, they have higher prediction accuracy (González-Camacho et al., 2018).

In recent years, deep learning (DL) has achieved great success in natural language processing, image recognition, and content generation (Otter et al., 2020; Li, 2022; Liu et al., 2021). With the introduction of artificial intelligence into the scientific field, many important discoveries have been made (Wang et al., 2023a). The Alphafold2 paper presented a DL calculation method for the first time to predict protein structures with atomic precision (Jumper et al., 2021). Liu and Wang (2017) proposed a DL model for predicting GEBV using convolutional neural networks (CNNs). The prediction accuracy of their DL model is greater than that of RRBLUP, Bayesian LASSO, and BayesA models. One study compared the genomic prediction accuracy of GBLUP, light gradient boosting machine (LightGBM), support vector regression (SVR), and DL (Wang et al., 2023b). The above research results show that deep neural network genomic prediction (DNNGP) outperforms most existing GS algorithms. The deep learning model performed better than the Bayesian and RRBLUP GS models, regardless of the wheat dataset size (Sandhu et al., 2021). SoyDNGP is a DL model for soybean trait prediction (Gao et al., 2023). It accurately predicts complex traits and shows robust performance across different sample sizes and trait complexity. Transformer-based GPformer is robust and stable to hyperparameters and can generalize to multiple species (Wu et al., 2024). Montesinos-López et al. (2021) reviewed the application of DL methods in GS and summarized the pros and cons of DL methods. The main pros of DL include: (1) DL models can capture non-additive effects and complex interactions among genes; (2) DL models can effectively handle multimodal data; (3) The DL architecture is very flexible and contains various modules. DL methods in GS have some defects: (1) DL is a black-box model and is not helpful for inference and association studies; (2) These models are more prone to overfitting than traditional statistical models; (3) Proper DL models require a very complex tuning process that relies on many hyperparameters. In general, deep learning algorithms are able to capture nonlinear patterns more effectively than traditional linear algorithms.

Through continuous research, DL for GS has revealed its advantages over other machine learning. DNNGP has the advantages of a simple model, high phenotypic prediction accuracy, and wide species adaptability. However, DNNGP requires hyperparameter tuning for each phenotype to achieve optimal performance. DNNGP is based on CNN, and the convolution kernel size is its crucial parameter. Since DNNGP is a serial structure, there can only be one kind of convolution kernel at one position. The serial structure causes an “information bottleneck”, and much information is lost in this calculation step (Tishby and Zaslavsky, 2015). Determining the convolution kernel size is a time-consuming and computationally intensive task. Convolutional synchronization has succeeded in the image field (Szegedy et al., 2015). It enables deeper neural networks and higher model prediction accuracy without time-consuming hyperparameter tuning. The data were simultaneously convolved with 1×1, 3×3, 5×5, and 7×7 convolutions to minimize information loss. The convolutional parallel structure increases the “width” of the model.

Different phenotypes have different prediction difficulties. Multiple studies show that the prediction accuracy of simple traits does not exceed 0.8 (Heffner et al., 2011; Heslot et al., 2012). The prediction accuracy of complex agronomic traits remains around 0.3. In many phenotype predictions, the prediction accuracy of DNNGP does not reach 0.8. This paper introduces convolution parallel technology into GS and adjusts it to adapt to one-dimensional convolution. This GS method is named parallel neural network for genomic selection (PNNGS). We develop PNNGS to improve the GS prediction accuracy further. To increase the stability of predictions, we introduce clustering algorithms and stratified sampling. The network architecture of PNNGS is similar to that of DNNGP, in which the convolutional layer is changed to a parallel convolutional layer. Each convolution branch has a different convolution kernel. To reduce the overfitting of PNNGS, we introduce residuals on each branch. We train PNNGS with four different loss functions. In the trait prediction of rice, sunflower [*Helianthus annuus* L.], wheat, and maize, the prediction accuracy of PNNGS is significantly greater than that of DNNGP, demonstrating convolutional parallelization’s effectiveness in GS. PNNGS can automatically obtain the optimal convolution size when simultaneously passing through convolution kernels of multiple sizes. It significantly reduces hyperparameter tuning effort. The prediction accuracy of PNNGS for most phenotypes is close to or exceeds 0.8, which meets the needs of practical applications. Through clustering algorithms, the plants are divided into different clusters. We find that wheat is an imbalanced dataset. Plants located in small clusters reduce the prediction accuracy of the phenotype. Reducing data imbalance is an important method to improve GS prediction accuracy.

## 2 Materials and methods

### 2.1 Plant materials

In this paper four public plant datasets have been analyzed. These datasets contain gene files and phenotype files. The corresponding plant phenotype is predicted through genomic data.

#### 2.1.1 Rice44k dataset

The Rice44k dataset comprises 413 inbred rice accessions collected from 82 countries (Zhao et al., 2011). These rice varieties were measured by 44k chips, and 36,901 SNP variants were obtained. Minor-allele frequency (MAF), missing call rate (MCR), and heterozygosity are three indicators for filtering sites in the literature (Thongda et al., 2020). Typical filter conditions are MAF > 0.05, MCR< 0.2, and heterozygosity< 0.05 (Zhang et al., 2022), which can filter out more than half of low-quality sites. Other thresholds for filtering sites, such as MAF > 0.01 or MAF > 0.1, have been applied in the literature (Backman et al., 2021; Liao et al., 2017). We filter rice SNP sites according to MAF > 0.05 and MCR< 0.2, and 33,163 SNPs are retained in the gene file. There are 34 phenotypes included in the Rice44k dataset. In this paper, we will investigate six of these phenotypes: flag leaf length (FLL), leaf pubescence (LP), panicle length (PL), plant height (PH), seed number per panicle (SNPP), and seed surface area (SSA).

#### 2.1.2 Sunflower1500k dataset

Marco Todesco et al. (2020) resequenced 1,506 wild sunflower strains from 3 species (*Helianthus annuus*, *Helianthus petiolaris* and *Helianthus argophyllus*). We only researched 614 samples of *Helianthus annuus*. Sunflower1500k is a large dataset containing 15,697,385 SNP sites and 87 traits. The number of SNPs filtered by MAF > 0.05 and MCR< 20% sites was 7,902,178. We randomly selected 30,179 sites and conducted the following research based on this gene file. This paper focuses on six traits, namely, flower head diameter (FHD), leaf perimeter (LPE), primary branches (PB), stem color (SC), stem diameter at flowering (SDF), and total RGB (TR). To distinguish it from leaf pubescence, the abbreviation of leaf perimeter is LPE.

#### 2.1.3 Wheat33k dataset

The Wheat33k dataset contains 2000 Iranian bread wheat landraces from the CIMMYT wheat gene bank (Crossa et al., 2016). It is a dataset with a relatively large sample size. Wheat33k contains 33709 markers and 8 phenotypes. Due to the high quality of the loci, we did not filter the gene files. Grain hardness (GH), grain length (GL), grain protein (GP), grain width (GW), thousand-kernel weight (TKW), and test weight (TW) are the six phenotypes focused on in this paper. The original literature describes the heritability of these six phenotypes as 0.839, 0.881, 0.625, 0.848, 0.833, and 0.754.

#### 2.1.4 Maize50k dataset

The Maize50k dataset contains genotype data from 282 inbred association panels (Cook et al., 2012). After discarding some nonconvertible sites, Maizi50k contained 50925 SNP sites. Through the same filter criteria, the number of SNPs is 45562. The phenotype file contains 285 trait/environment combinations for 57 traits collected between 2006 and 2009. The genotypic and phenotypic data are obtained from *Panzea* datasets. The phenotype file name of Maizi50k is maize282NAM-15-130212. We download it from the Internet (http://cbsusrv04.tc.cornell.edu/users/panzea/download.aspx?filegroupid=9). The phenotype file contains 16 environments. Days to silk (DS) is a phenotype with six environments. Detailed descriptions of these environments are provided in **Supplementary Table S1**. The codes for the six maize environments are 06CL1, 065, 26M3, 07CL1, 07A, and 06PR. Through the Maize50k dataset, we study the PNNGS performance in predicting multi-environment phenotypes.

### 2.2 PNNGS architecture

**Figure 1:**
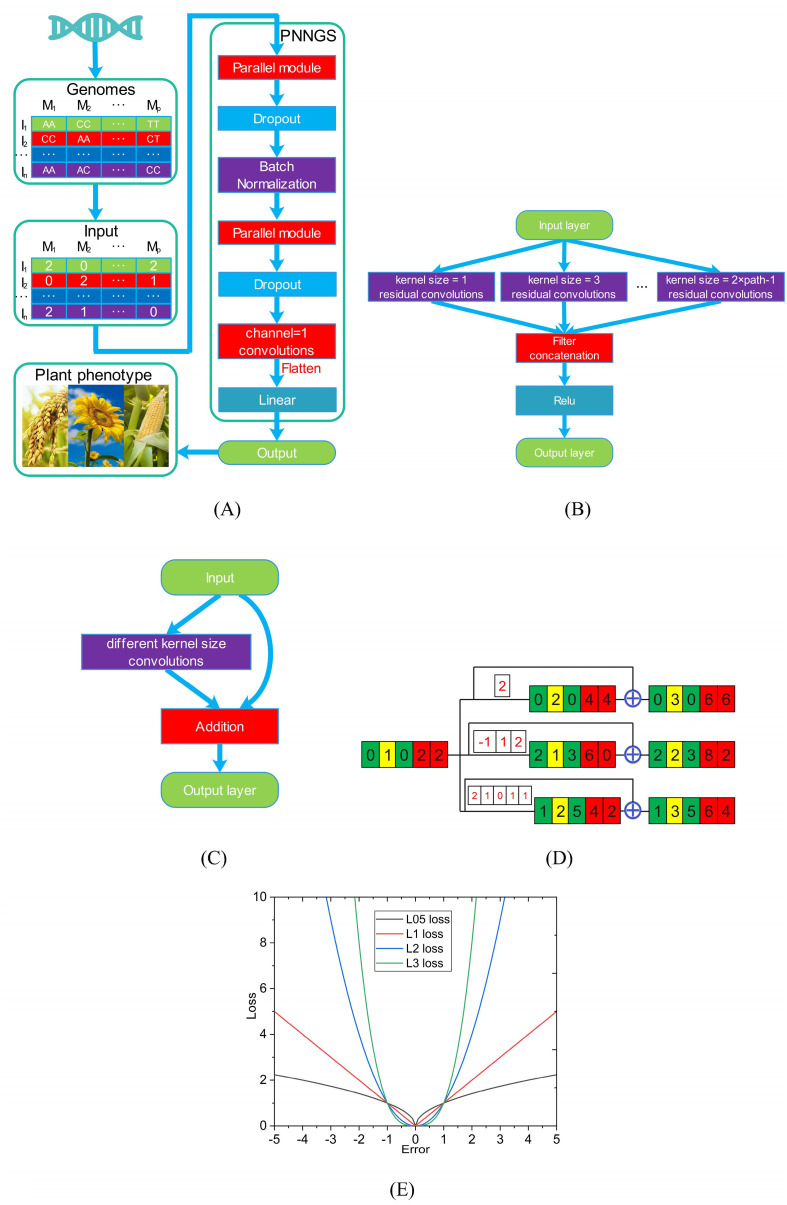
Schematic diagram of PNNGS. Plant phenotypes and genome sequences are collected. Homozygous dominant, heterozygous, and homozygous recessive are coded as 2, 1, and 0. The input and output of PNNGS are the genome matrix and plant phenotype, respectively. The parallel module includes multiple convolutions of different kernel sizes. **(A)** architecture of PNNGS; **(B)** structural details of the parallel module; **(C)** residual convolution with different kernel sizes; **(D)** PNNGS calculation process with three branches; **(E)** four L*p* loss functions. PNNGS, a parallel neural network for genomic selection.

The plant breeding values in GS are estimated by thousands or tens of thousands of SNP sites distributed throughout the genetic material. The first step is to collect plant genome sequences and phenotypes (**Figure 1A**). Since the collected phenotypes often have some flaws, data cleaning is required. Typical data cleaning includes removing outliers, imputing missing data, and discarding plants. The genotypes of diploid plants are divided into three types: homozygous dominant, heterozygous, and homozygous recessive, which are typically coded as 0, 1, and 2 (Lippert et al., 2011). Each wheat allele is recorded as 1 (present) or 0 (absent). These genotype encoding methods are adopted in this paper. The rows and columns of the input matrix are samples and SNPs, respectively. The number of samples in current GS application scenarios is generally several hundred (Hickey et al., 2017). With the advancement of sequencing technology, SNP sequencing length has reached millions or even tens of millions. The number of SNPs is four to five orders of magnitude greater than the number of samples. The situation mentioned above is the famous “*p*>>*n*” problem in the GS field, where *p* represents the number of SNPs and *n* represents the number of samples (Yan and Wang, 2023).

The right side of **Figure 1A** shows the architecture of PNNGS. PNNGS consists of a parallel module, a dropout layer, a batch normalization layer, a parallel module, a dropout layer, and a linear layer in sequence. The dropout layer can reduce model overfitting and alleviate the “*p*>>*n*” problem, and the dropout rate is set to 0.5. A large dropout rate can effectively resist overfitting. However, it requires the DL architecture to be quite robust. The batch normalization (BN) layer speeds up network training and convergence. It controls gradient explosion, prevents gradient vanishing, and prevents overfitting. Modern neural networks generally add BN to improve performance (Ioffe and Szegedy, 2015). The data needs to be flattened before entering the linear layer. The output of the linear layer is the prediction of the plant phenotype. PNNGS realizes the transformation from plant genotype to phenotype.

The parallel module contains multiple parallel residual convolutions, which is the main innovation of this paper (**Figure 1B**). The kernel sizes of the first and second paths are 1 and 3, respectively. The kernel size of the *n* ^th^ path is 2*n*-1. Then, the calculation results of all parallel branches are concatenated. To increase the nonlinear representation capability of PNNGS, we pass the data through the rectified linear activation (Relu) layer and obtain the output. In image convolution, we generally use two layers of 3×3 convolutions instead of one layer of 5×5 convolution. However, this technique does not work here. In one-dimensional convolution, the convolution parameters are proportional to the kernel size. We can directly operate using large kernel-size convolutions. The calculation process of the parallel module can be expressed through a mathematical formula:


y=Relu(concatenate(f(x,1),f(x,3),⋯,f(x,2n−1)))

where *x* is the input and *y* is the output. Function *f* is a one-dimensional residual convolution operation. Its second parameter is the kernel size. The convolution outputs are concatenated in the channel dimension. Relu is the most frequently used activation function in deep learning models. If the function receives any negative input, it returns 0. However, for any positive value, it returns itself.

The residual convolution is the sum of the input and the convolution corresponding to the input (**Figure 1C**). The residual convolutions in different paths contain different kernel sizes. Compared with simple convolution operation, residual convolution improves model fitting ability and anti-overfitting ability. **Figure 1D** shows the PNNGS calculation process with three branches. The input is [0, 1, 0, 2, 2]. The convolution kernels of the three branches are [2], [-1, 1, 2], and [2, 1, 0, 1, 1]. To keep the output the same size as the input, we pad the input with appropriate zeros on both sides. Through the convolution operation, the outputs are [0, 2, 0, 4, 4], [2, 1, 3, 6, 0], and [1, 2, 5, 4, 2] respectively. After further residual calculation, the final output is [0, 3, 0, 6, 6], [2, 2, 3, 8, 2], and [1, 3, 5, 6, 4]. With a simple convolution operation, the output will be [2, 1, 3, 6, 0]. Apparently, PNNGS performs more diverse computations on the input.

The optimizer for PNNGS is Adam. The learning rate and weight decay are set to 0.001 and 0.1, respectively. Weight decay is essentially an L2 regularization coefficient. Training PNNGS is challenging since the loss function cannot be set to the Pearson correlation coefficient. The L*p* loss function is the most popular in the field of machine learning. *p* is a parameter that adjusts the sensitivity to outliers. When *p* is small, the model is robust. Conversely, it makes better predictions for outliers. Common L*p* loss functions in the literature are L1 and L2. The L1 and L2 losses are the mean absolute error (MAE) and mean squared error (MSE), respectively. We train PNNGS based on L05, L1, L2, and L3 loss functions (**Figure 1E**). L05 refers to the L*p* loss function with *p* = 0.5.


$$Lp\;Loss=\sum_{i=1}^{n}{\left(\left|y_{i,true}-y_{i,pred}\right|\right)}^{p}$$

The sample size is *n*. *y_i_*,*_true_* and *y_i_*,*_pred_* are the true phenotype and predicted phenotype of the *i* ^th^ individual, respectively. We tried adding more layers to PNNGS, which only reduced the L*p* loss but not the Pearson correlation coefficient.

PNNGS is a fairly small model for the popular large models. More details about PNNGS are available in the source program.

### 2.3 Four other GS models

Four GS models (RRBLUP, RF, SVR, and DNNGP) have been compared with PNNGS for phenotypic prediction accuracy. These four GS models have the characteristics of simple principle, stable performance, and wide application.

#### 2.3.1 RRBLUP model

The RRBLUP model is based on the best linear unbiased prediction model (Rice and Lipka, 2019). The BLUP model is described as follows:


$$y_{i}=\mu +\sum_{k=1}^{q}x_{ik}\beta_{k}+\epsilon_{i}$$

where *μ* is the phenotypic mean. *x_ik_* is the genotype of the *k* ^th^ site of the *i* ^th^ individual. *q* represents the number of SNP sites. *β_k_* is the estimated random additive SNP site effect at the *k* ^th^ site, and *ϵ_i_* is the residual error term. *y_i_* is the phenotype of the *i* ^th^ individual.

The loss function of RRBLUP is:


$$Loss\left(y_{true},\;y_{pred}\right)=\frac{1}{n}\sum_{k=1}^{q}{\left(y_{i,true}-y_{i,pred}\right)}^{2}+\lambda \sum_{k=1}^{q}\beta_{k}^{2}$$

where $\lambda \overset{p}{\underset{k=1}{\sum }}\beta_{k}^{2}$ is the ridge regression penalty, which reduces the value range of *β_k_*. *λ* is a hyperparameter that controls the intensity of the penalty. Our goal is to minimize the loss.

The BLUP with penalty term is RRBLUP. Compared with BLUP, the stability and prediction accuracy of RRBULP are improved simultaneously.

#### 2.3.2 RF model

RF is a popular supervised machine learning method for classification and regression. It combines the predictions of multiple decision trees into a single overall prediction (Annicchiarico et al., 2015). Training a random forest means training each decision tree independently. The principle of RF is that the variance of each decision tree will help avoid overfitting. It is easy to overfit when training a single decision tree on the entire training set. Random forest regression (RFR) is an ensemble learning method. RFR is widely used in the GS field, and its prediction accuracy and generalization are competitive (Blondel et al., 2015).

#### 2.3.3 SVR model

SVR is a machine-learning technique for regression tasks. It is a variant of SVM designed to predict continuous values, making it suitable for quantitative trait prediction. SVR identifies the “margin” around the predicted regression line. Its goal is to fit a straight line within this margin while minimizing the prediction error. SVR is robust to outliers because it focuses primarily on data points near the edges instead of relying heavily on all data points (Üstün et al., 2005). It is beneficial in dealing with nonlinear relationships and can be adapted to various problem domains by selecting kernel functions (Wu et al., 2009). In wheat GS, a nonlinear RBF kernel is an optimal choice for SVR (Long et al., 2011).

#### 2.3.4 DNNGP model

DNNGP is a recent deep-learning algorithm for GS. It clarifies that BN, early stopping, and Relu are three effective techniques for GS. The architecture of DNNGP is simple yet effective, as it balances sample size and network depth well. It is the first deep-learning algorithm that clearly outperforms LightGBM and SVR in the GS domain. DNNGP contains three CNN layers, one BN layer, and two dropout layers. It is serial and has no branches. Compared with DNNGP, PNNGS chooses to increase the width of the network instead of the depth. Since the sample size is only a few hundred, neural networks with more than five layers are prone to overfitting (Zou et al., 2019).

### 2.4 Evaluation criteria

The Pearson correlation coefficient is applied as the evaluation criterion for the GS model. The Pearson correlation coefficient ranges from -1 to 1. In most cases, its value ranges from 0 to 1 in the GS model. The GS model makes a perfect prediction when the Pearson correlation coefficient is 1. When the Pearson correlation coefficient is 0, the phenotype predicted by the GS model is linearly independent of the observed phenotype. Since the value range of the Pearson correlation coefficient is fixed, it is easy to compare the performance of the GS model on different phenotypes. Compared with MAE and MSE, the Pearson correlation coefficient is a more appropriate GS evaluation criterion. When the GS model predicts the phenotype of all plants as an average, MAE and MSE still give an excellent score for this predictive measure. In this case, the Pearson correlation coefficient is 0, indicating that the GS model for predicting the phenotypic mean is the worst. The Pearson correlation coefficient is the most popular evaluation criterion in the GS field (Akdemir et al., 2015).

The normalized root mean square error (NRMSE) is calculated as the root mean square error divided by the range of the observations, expressed as a percentage. The range of the observations is the difference between the maximum and minimum values of the observed data. The value range of NRMSE is [0, +inf). NRMSE is our secondary evaluation criterion.

### 2.5 Phenotype distribution pattern

**Figure 2:**
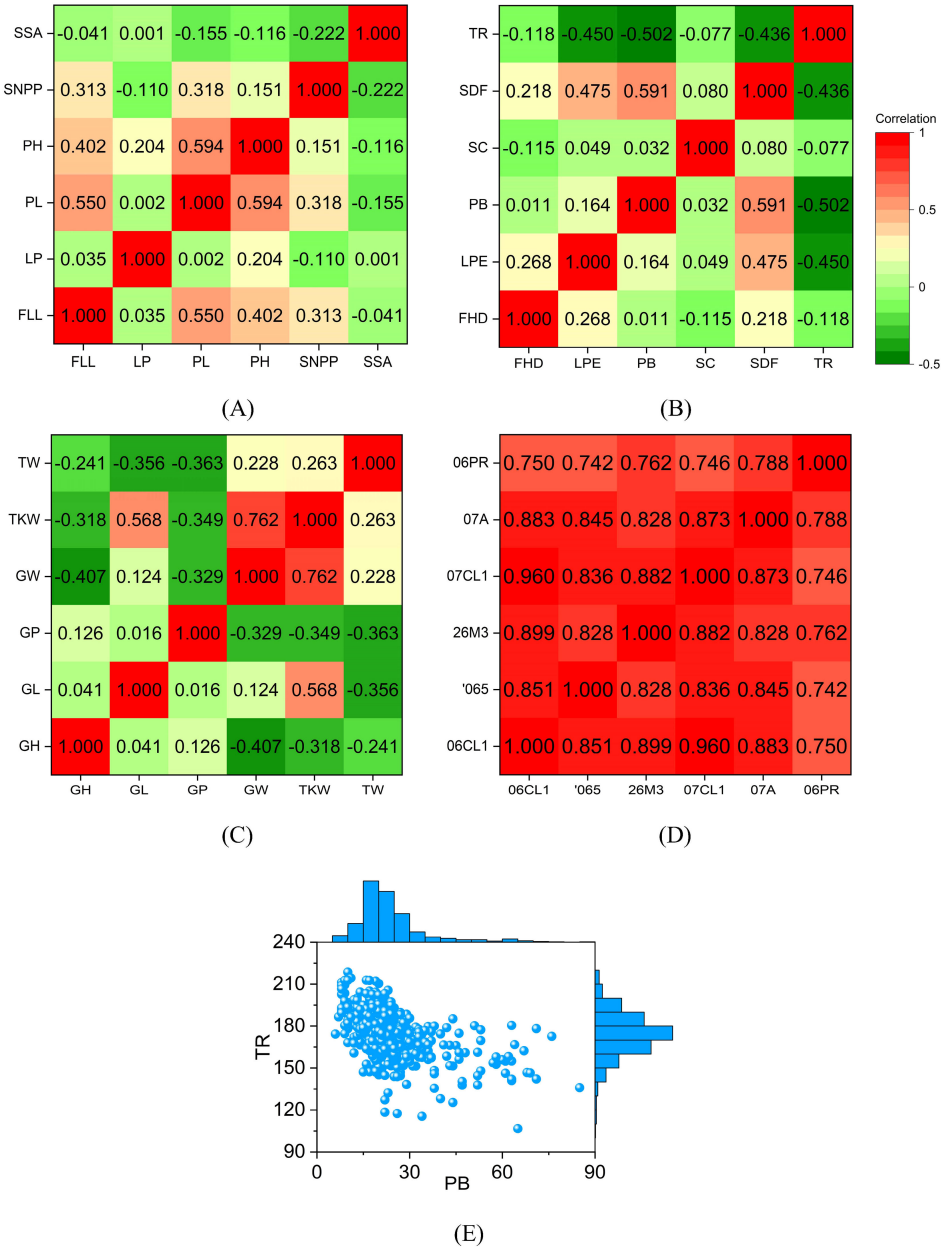
Correlations between different phenotypes. The value in the grid is the Pearson correlation coefficient of the two phenotypes. The correlations between different phenotypes are small. The correlation coefficient of the same phenotype under different environments is large. The distribution of PB is scattered, and the distribution of TR is relatively concentrated. **(A)** the Pearson correlation coefficient between different rice phenotypes; **(B)** the Pearson correlation coefficient between different sunflower phenotypes; **(C)** the Pearson correlation coefficient between different wheat phenotypes; **(D)** the Pearson correlation coefficient of maize days to silk under different environments; **(E)** the Distribution of sunflower PB and TR. In rice, FLL, flag leaf length; LP, leaf pubescence; PL, panicle length; PH, plant height; SNPP, seed number per panicle; SSA, seed surface area. In sunflower, FDD, flower head diameter; LPE, leaf perimeter; PB, primary branches; SC, stem color; SDF, stem diameter at flowering; TR, total RGB. In wheat, GH, grain hardness; GL, grain length; GP, grain protein; GW, grain width; TKW, thousand-kernel weight; TW, test weight. 06PR, 07A, 07CL1, 26M3, 065, and 06CL1 are the codes for the six maize environments.

To predict phenotypes more accurately, we need to analyze phenotypic distributions. The correlations among the six rice phenotypes are relatively weak. The Pearson correlation coefficients between most phenotypes are less than 0.3, meaning they are linearly independent (**Figure 2A**). The Pearson correlation coefficient between PH and PL is 0.594. PH and PL increase with increasing mid-season temperature, which results in a strong correlation between them (Kovi et al., 2011). The prediction accuracy of GS for highly correlated phenotypes is similar. The Pearson correlation coefficient between FLL and PL is 0.55, indicating a positive correlation. Flag leaf plays a vital role in providing photosynthetic products to grains. Plants with long FLL elongate PL, resulting in increased grain number per ear (Rahman et al., 2013). When PL increases, PH and FLL increase with considerable probability.

The Pearson correlation coefficients between most sunflower phenotypes are less than 0.3 (**Figure 2B**). There is a strong positive correlation between PB and SDF, as ethrel can increase both PB and SDF (Kumar et al., 2010). TR is a unique phenotype because it is negatively correlated with other phenotypes. TR is defined as the sum of RGB values of leaf color. TR increases significantly when the red channel signal of leaves is enhanced (Chen et al., 2020). Therefore, a significant TR indicates yellow leaves and a small TR means green leaves. If TR is significant, the photosynthesis efficiency of the leaves will be low, and the plant growth will be poor. TR has the strongest negative correlation with PB.

The Pearson correlation coefficients between wheat phenotypes are mostly less than 0, indicating that most phenotypes are negatively correlated (**Figure 2C**). Wide grains reduce grain hardness, which is consistent with the mechanical properties of the material. Therefore, the Pearson correlation coefficient between GH and GW (-0.407) is much less than 0. GL and TKW are significantly positively correlated, and their Pearson correlation coefficient is 0.568. GP is negatively correlated with GW, TKW, and TW, indicating that there is a contradiction between wheat yield and grain protein content. The bad news is that it is difficult to obtain wheat varieties that are both high in yield and high in protein. The Pearson correlation coefficient between GW and TKW is 0.762, which indicates that the key to increasing wheat yield is to increase grain width.

Different from rice, sunflower, and wheat, we calculate the Pearson correlation coefficient of the same phenotype in maize under different environments. The correlation coefficients between phenotypes are all greater than 0.7 (**Figure 2D**). The Pearson correlation coefficient between 06CL1 and 07CL1 reaches 0.96, which means that we can predict the DS in 07CL1 through the DS in 06CL1. The difference between 06CL1 and 07CL1 is minimal, and their difference is one year in planting date. The correlation coefficients between the DS in 06PR and the DS in other environments range from 0.7 to 0.8. 06PR is the only winter environment among the six environments, and the other five are all summer. The above analysis results show that the correlation between the same phenotype in different environments is much more significant than the correlation between different phenotypes in the same environment. Since the correlation coefficients between different phenotypes are small, genomic prediction is required for each phenotype.

There are 570 sunflowers with both PB and TR. Their distribution is shown in **Figure 2E**. PB has a long-tailed, right-skewed distribution. The maximum, minimum, mean, and standard deviation of PB is 85, 6, 23.1, and 11.4, respectively. TR approximately satisfies the normal distribution, and its maximum, minimum, mean, and standard deviation values are 218.7, 106.7, 174.2, and 15.9, respectively. Compared with PB, TR has a larger span. However, TR is more concentrated. The coefficient of variation is the ratio of the standard deviation to the mean and is a measure of the dispersion of a data set. The variation coefficients of PB and TR are 0.49 (= 11.4/23.1) and 0.09 (= 15.9/174.2) respectively. A small coefficient of variation means that the data are compact. Therefore, PB is more dispersed than TR.

## 3 Results

### 3.1 Selection of the number of parallelism

The computing platform is Intel(R) i7-8700 CPU, RTX 3090 GPU, 32 GB RAM, and Windows 10. PNNGS and DNNGP are implemented through torch 1.7. RRBLUP, RF, and SVR are implemented based on scikit-learn 1.3. All calculations are performed in Python, and the source code is open.

For PNNGS, determining the number of parallelism is the primary task. The number of parallelism is a hyperparameter, and its specific value is not presented in the PNNGS architecture. We need to do a grid search experiment to determine the number of parallelism, and the experimental results are presented in **Table 1**. The number of parallels could be 2, 3, 4, 5, 6, 7, and 8. The experimental phenotypes are SNPP in rice, FHD in sunflower, GH in wheat, and DS_065 in maize. For SNPP, the prediction accuracy first increases and then decreases with the increase of parallelism. When the parallel number is 4, the prediction accuracy of SNPP is the highest, which is 0.664. If the parallel number is inappropriate, the phenotype prediction accuracy will drop by 0.014. FHD, GH, and DS_065 show similar change patterns. The optimal parallel numbers for FHD, GH, and DS_065 are 6, 4, and 3, respectively.

**Table 1:** Phenotype prediction accuracy with different numbers of parallelism.

Parallel number	2	3	4	5	6	7	8
SNPP	0.651	0.656	**0.664**	0.652	0.657	0.655	0.650
FHD	0.675	0.665	0.673	0.669	**0.675**	0.674	0.671
GH	0.690	0.691	**0.702**	0.700	0.699	0.695	0.698
DS_065	0.817	**0.829**	0.828	0.821	0.804	0.810	0.813

The calculation model is PNNGS. SNPP, FHD, GH, and DS_065 represent the phenotypes of rice, sunflower, wheat, and maize, respectively. The best predictions are in bold.

In the following phenotypic predictions, the parallel numbers of rice, sunflower, wheat, and maize phenotypes are 4, 6, 4, and 3, respectively. We did not perform a grid search for each phenotype. Due to the stochastic nature of neural networks, the calculation results of PNNGS may fluctuate slightly. In repeated calculations, the optimal parallel number of PNNGS may be slightly different from **Table 1**. However, it has little impact on the prediction results.

### 3.2 PNNGS prediction accuracy for phenotypes

We utilized PNNGS to predict previously analyzed phenotypes. The rice, sunflower, and wheat phenotypes were designed to test the predictive ability of PNNGS for different phenotypes. Six environmental phenotypes of maize were predicted to obtain the performance of PNNGS under different environments. To reduce the impact of dataset partitioning, we introduce ten-fold cross-validation in this study. The average of the ten Pearson correlation coefficients for the phenotype is regarded as the final prediction accuracy of PNNGS for the phenotype. Through NRMSE, we know the difference between the predicted value and the true value. Therefore, NRMSE also evaluates the effect of model prediction. We simultaneously applied RRBLUP, RF, SVR, and DNNGP to predict these phenotypes and compare their prediction ability with PNNGS.

**Figure 3:**
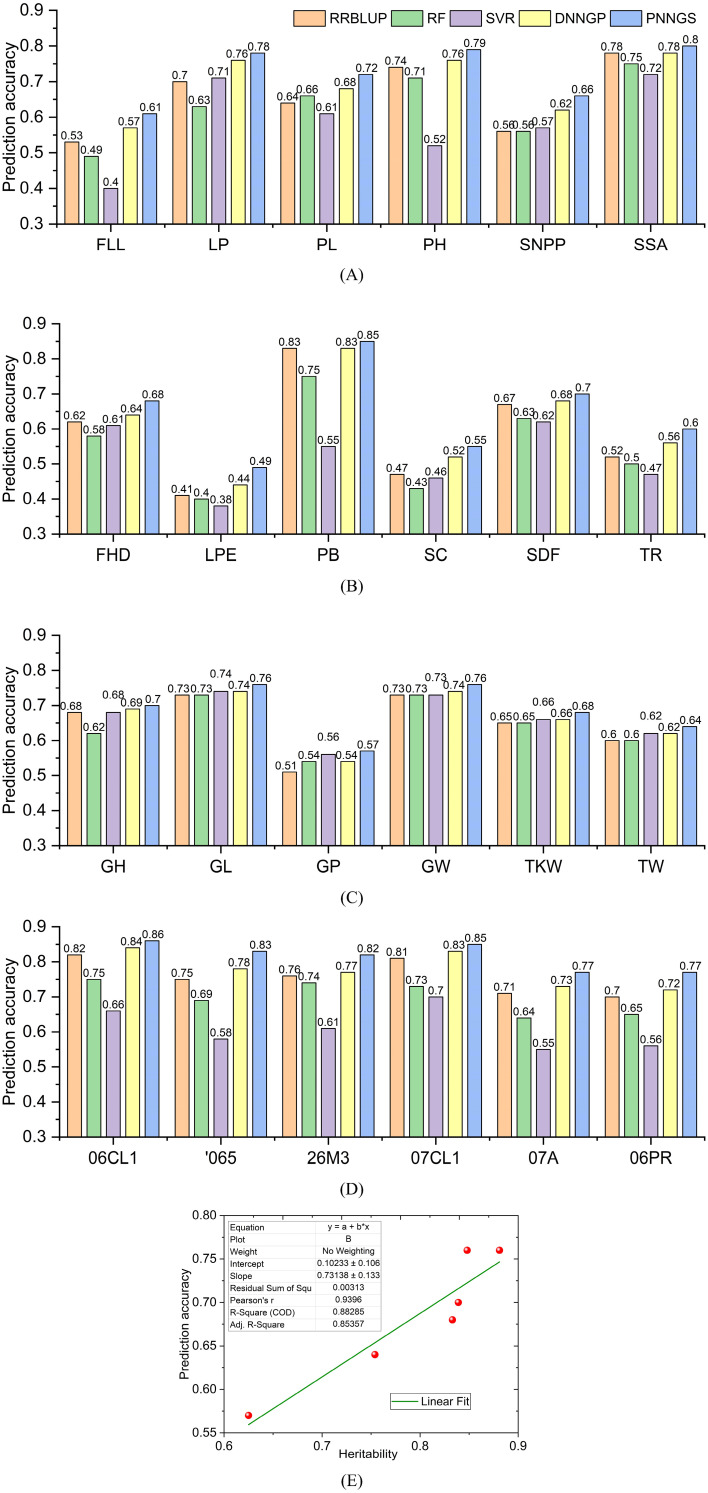
Prediction accuracy of plant phenotypes by different algorithms. DNNGP outperforms RRBLUP, RF, and SVR. PNNGS is 0.01 to 0.05 more accurate than DNNGP in prediction. **(A)** rice; **(B)** sunflower; **(C)** wheat; **(D)** maize; **(E)** the relationship between heritability and prediction accuracy. RRBLUP, the ridge-regression best linear unbiased prediction; RF, random forests; SVR, support vector regression; DNNGP, deep neural network genomic prediction.

In FLL, LP, PL, PH, SNPP, and SSA predictions, RRBLUP, RF, and SVR are competitive (**Figure 3A**). The prediction accuracy of DNNGP is greater than or equal to that of RRBLUP, RF, and SVR. DNNGP can obtain robust phenotype predictions on different datasets. Compared with serial DNNGP, parallel PNNGS achieves higher prediction accuracy. Among the six phenotypes, the prediction accuracy of PNNGS was higher than that of DNNGP by 0.04, 0.02, 0.04, 0.03, 0.04, and 0.02. As the prediction accuracy of DNNGP increases, the prediction accuracy improvement of PNNGS decreases. PNNGS can significantly improve the prediction accuracy of complex phenotypes, which is of great significance in practical applications.

The prediction accuracy of the six sunflower phenotypes is presented in **Figure 3B**. The prediction accuracies of the five algorithms in LPE, SC, and TR do not exceed 0.6, indicating that these three phenotypes are difficult to predict. PB is an easy-to-predict phenotype because the prediction accuracy of RRBLUP, DNNGP, and PNNGS for it is all over 0.8. The prediction difficulty of PB and TR is consistent with the analysis results in Section 2.5. Among the six sunflower phenotype predictions, DNNGP and PNNGS are in the leading position. The average prediction accuracy of PNNGS is 0.033 higher than that of DNNGP.

Among all phenotypic predictions for wheat, PNNGS is the best-performing algorithm (**Figure 3C**). The wheat case is significantly different from the other three cases. Rice, sunflower, and maize are diploid, while common wheat is an allohexaploid. Wheat has the largest sample size, reaching two thousand. PNNGS has the greatest improvement in GP prediction accuracy and the least improvement in GL prediction accuracy. Meanwhile, GP has the lowest average prediction accuracy, while GL has the opposite. In the prediction of six wheat phenotypes, the average prediction accuracy of PNNGS was 0.02 higher than that of DNNGP. Among the four crops, PNNGS provided the smallest improvement in wheat phenotypic prediction. The performance of PNNGS does not improve as the sample size increases. Each SNP in diploid crops has three types: homozygous dominant, heterozygous, and homozygous recessive. However, each SNP in wheat has only two types: present and absent. With the same number of SNPs, wheat genotypes contain less information, which may make wheat phenotype more difficult to predict. Increasing the sample size can improve the prediction accuracy of all algorithms. However, 2,000 samples do not make PNNGS significantly ahead of other algorithms.

Different algorithms have similar DS prediction accuracy for the six environments (**Figure 3D**). The algorithm rankings according to prediction accuracy are PNNGS, DNNGP, RRBLUP, RF, and SVR. The algorithmic ranking order does not change with the environment. The difference in phenotypic prediction accuracy of 06CL1 and 07CL1 by the same algorithm is approximately 0.01 because the correlation coefficient between 06CL1 and 07CL1 reaches 0.96. The prediction accuracy of DS is the lowest in the 06PR environment, and winter has a significant impact on maize growth. The algorithms in this paper only consider biological genome information and do not consider environmental factors. Strong interactions between genes and the environment can reduce the prediction accuracy of the GS algorithm. We can first predict the phenotype in an environment through GS. The calculated phenotypes are then used to predict phenotypes in other environments.

The average phenotypic prediction accuracies of RRBLUP, RF, SVR, DNNGP, and PNNGS in 24 calculation cases are 0.663, 0.632, 0.595, 0.687, and 0.718, respectively. RRBLUP outperforms two traditional machine learning algorithms, RF and SVR, indicating that the linear model is highly competitive in GS. DNNGP is an emerging deep-learning algorithm that exceeds the previous three models in phenotypic prediction accuracy and stability. PNNGS proposes a parallel architecture based on DNNGP, improving the prediction accuracy by 0.031 (= 0.718-0.687). The phenotypic calculation results verify the effectiveness of the parallel neural network.

**Figure 4:**
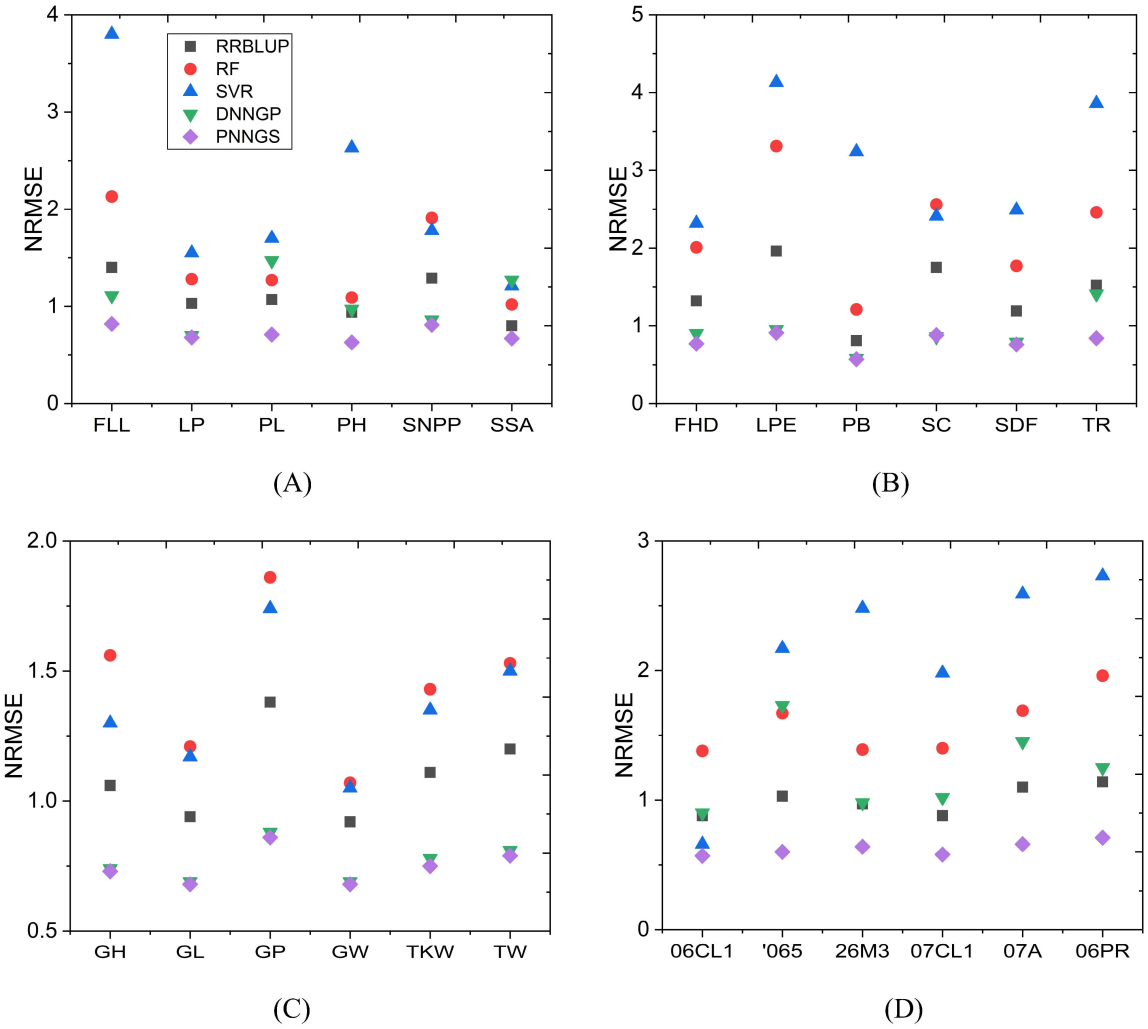
NRMSE of plant phenotypes by different algorithms. PNNGS ranks first in all phenotype predictions. **(A)** rice; **(B)** sunflower; **(C)** wheat; **(D)** maize. NRMSE, normalized root mean square error.

The NRMSE of phenotype prediction for rice, sunflower, wheat, and maize is shown in **Figure 4**. NRMSE is normalized and has no units. The NRMSE of RF and SVR is large, which indicates that the prediction errors of RF and SVR are the largest. DNNGP performs very well in sunflower and wheat. However, it performs poorly in maize. DNNGP does not perform as well on NRMSE as on the Pearson correlation coefficient. In the vast majority of phenotype predictions, RRBLUP ranks second or third. PNNGS ranks first in all phenotype predictions. In some phenotype predictions, DNNGP is very close to PNNGS. The greatest advantage of PNNGS is its stable performance. In all scenarios, PNNGS ranks first for both evaluation indicators, although sometimes the lead is not large.

The heritability of wheat phenotypes is presented in Section 2.1.3. Here, we plot the relationship between heritability and prediction accuracy (**Figure 3E**). There is a significant positive correlation between prediction accuracy and heritability. The fitting straight line is *y*=0.10233 + 0.73138*x*. *x* and *y* are heritability and prediction accuracy, respectively. *R* ^2^ (0.88285) is greater than 0.8, indicating that the linear fit is very appropriate. In general, traits with high heritability are easier to select in breeding, while traits with low heritability are more difficult to select in breeding. The conclusion about heritability can also be extended to: traits with high heritability have higher prediction accuracy, while traits with low heritability have lower prediction accuracy.

### 3.3 PNNGS prediction stability

**Figure 5:**
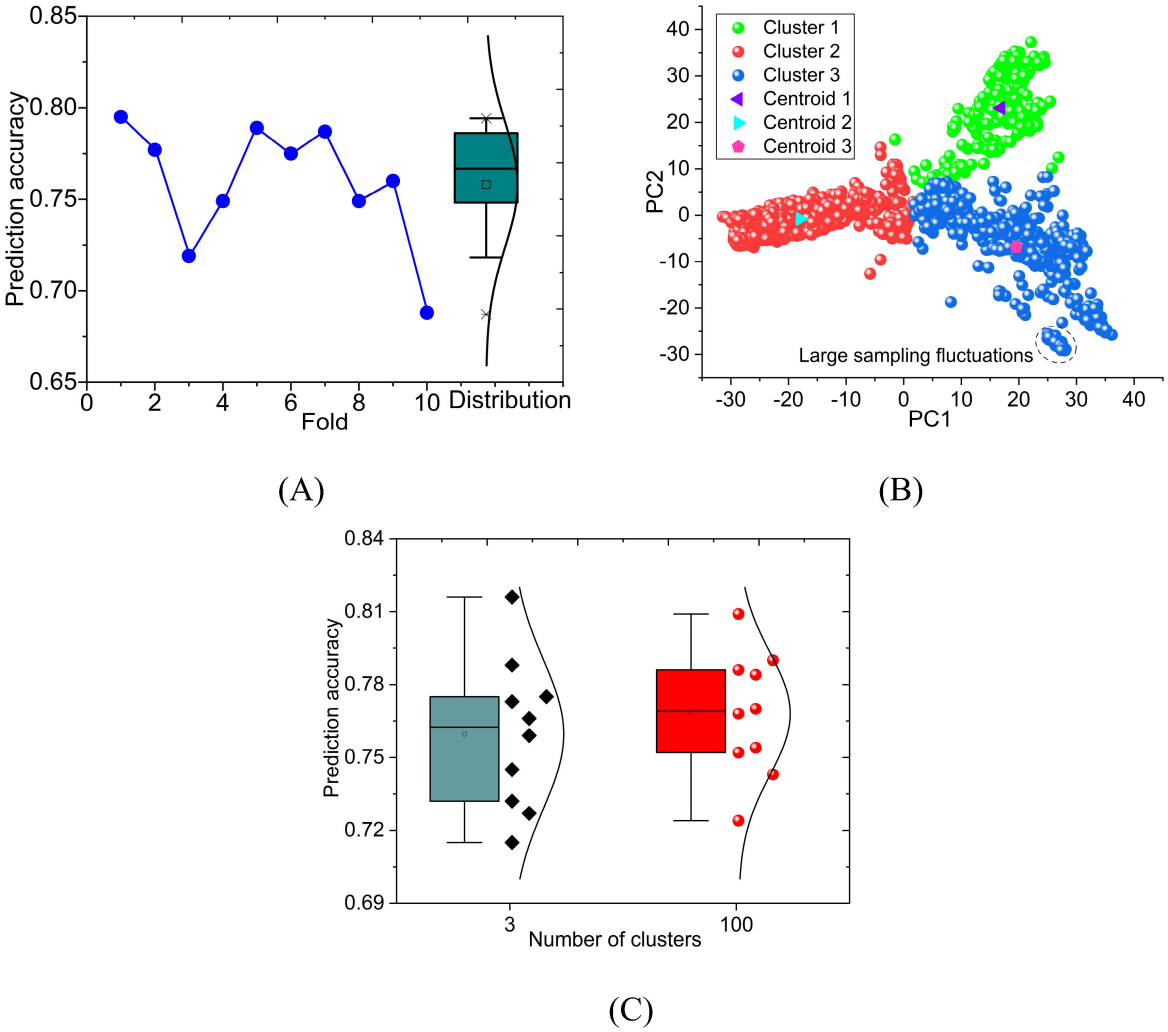
Ten-fold cross-validation calculation results of PNNGS. The calculated phenotype is the GL of wheat. The prediction accuracy of ten-fold cross-validation fluctuates widely. Phenotypic prediction accuracy has little correlation with the phenotypic difference between the training and the test sets. PCA is performed on genomic data. The data set is divided into three clusters through K- means. Stratified sampling analysis is performed based on clustering results. **(A)** prediction accuracy obtained by ten-fold cross-validation and its distribution; **(B)** PCA and K-means clustering; **(C)** calculation results for 3 clusters and 100 clusters. PCA, Principal component analysis.

The purpose of ten-fold cross-validation is to evaluate the predictive ability of the model more stably. However, the average prediction accuracy loses the prediction information of each fold. We plotted the prediction accuracy of PNNGS for each fold of GL (**Figure 5A**). The maximum and minimum prediction accuracy values of these ten folds are 0.795 and 0.688, respectively. The standard deviation of prediction accuracy is 0.032. The prediction accuracy fluctuates significantly at different folds. The difference in GL prediction accuracy (= 0.795-0.688) under different folds is more significant than the difference between different models (= 0.76-0.73), which means that there are some flaws in the current calculation.

Machine learning requires that the data in the training set and test set are independent and identically distributed. Since different varieties of plants do not affect each other, the data independence condition is generally met. If the sample size is large, the training and test sets obtained by random sampling are generally identically distributed. When the sample size is insufficient, random sampling may result in different distributions for the training and test sets. If the identical distribution cannot be satisfied, the prediction accuracy of machine learning in the test set will generally be lower than the prediction accuracy of the training set. Unfortunately, the sample size is only a few hundred or a few thousand in GS, and the above situation often occurs.

We performed principal component analysis (PCA) on the genomic data (**Figure 5B**). The component is set to 2, which makes it convenient to display the results. Intuitively, the data forms three clusters. K-means clustering is introduced, and its *n*-clusters are set to 3. Each plant can be classified into Cluster 1, Cluster 2, and Cluster 3 (**Supplementary Table S2**). The number of plants in Cluster 1, Cluster 2, and Cluster 3 are 251, 1025, and 724, respectively. There are a total of 2000 (= 251 + 1025 + 724) wheat plants with GL. The centroid coordinates of Cluster1, Cluster2, and Cluster3 are (16.8, 23.1), (-18.0, -0.8), and (19.5, -6.9), respectively. For the same dataset, we divided the dataset into 100 clusters through the same method (**Supplementary Table S3**). On average, each cluster has 20 samples. Since there are too many categories, it is not convenient to display them in figures.

The previous calculations are all based on random sampling. Along with clustering, stratified sampling is introduced. We again predict GL through stratified sampling and ten-fold cross-validation (**Figure 5C**). According to the results in **Figure 5A** and **Figure 5C**, the fitted normal distribution becomes more peaked as the number of clusters increases. It indicates that the standard deviation of the prediction results is gradually decreasing. When the number of clusters is 3 and 100, the standard deviations of the prediction results are 0.029 and 0.024, respectively. The standard deviations decrease by 0.003 (= 0.032-0.029) and 0.008 (= 0.032-0.024) respectively. The prediction stability of PNNGS is significantly improved at different folds. Another notable improvement is the increase in GL prediction accuracy. When the number of clusters is 1, 3, and 100, the GL prediction accuracy is 0.759, 0.760, and 0.768, respectively.

In **Figure 5B**, we marked a tiny cluster of closely spaced samples with dashed lines. We can only accurately predict the samples in this tiny cluster based on other samples in this tiny cluster. In random sampling, there is no guarantee that samples in tiny clusters will appear in the training set. In this case, the prediction accuracy is low, and the fluctuation is large. If we divide the data into 100 clusters and perform stratified sampling, some samples in the dashed line will definitely be in the training set. The quality of the training set data is improved. The phenotypic prediction accuracy became stable among different folds.

Since the training set is randomly sampled rather than stratified, it causes large fluctuations in prediction accuracy at different folds. Stratified sampling should not be performed based on phenotype. We need to perform PCA on the genomic data first. Plants are clustered through a clustering algorithm. Stratified sampling according to categories can reduce fluctuations in prediction accuracy, allowing us to evaluate the model more objectively.

## 4 Discussion

The main factor currently plaguing the application of GS is its low prediction accuracy. The introduction of deep learning improves the GS prediction accuracy. However, the prediction accuracy of complex traits still cannot meet the needs of practical agricultural applications. Insufficient sample size is the most important factor restricting the further improvement of deep learning for GS. Insufficient samples destroy the identical distribution of the training and test sets. High-quality samples are the key to solving all the above problems.

**Figure 6:**
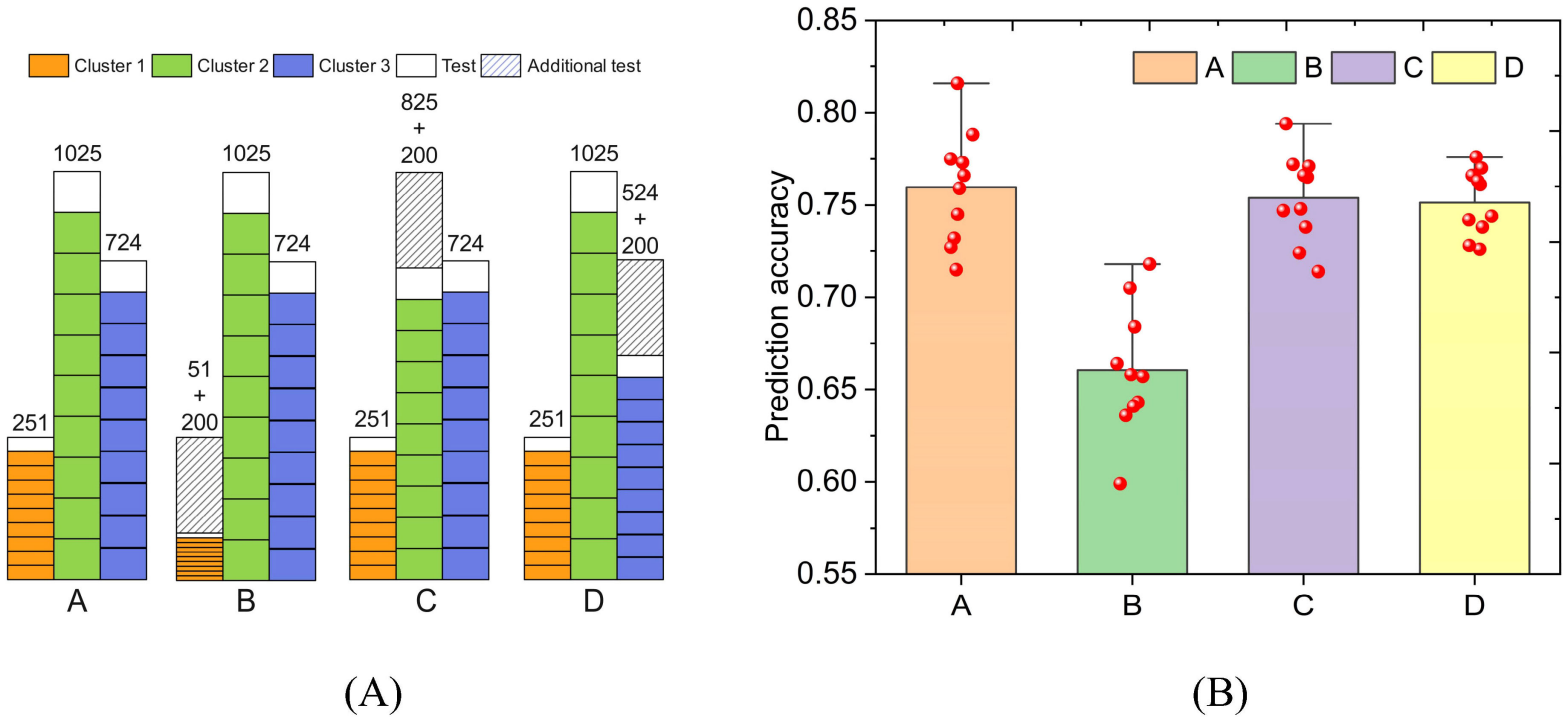
The impact of reducing samples in different clusters on prediction results. The predicted phenotype is wheat GL. Cluster 1 has the least samples. The phenotype prediction accuracy decreased the most when reducing the samples in cluster 1. **(A)** four schemes to divide the training set and test set; **(B)** Prediction results of four schemes.

We designed four schemes to establish PNNGS (**Figure 6A**). The prediction object is wheat GL. In Scheme A, all data were subjected to stratified sampling and ten-fold cross-validation. In scheme B, we randomly select 200 samples from Cluster 1 as an additional test set. Stratified sampling and ten-fold cross-validation were performed on the remaining samples. These additional test sets are also used to test the prediction accuracy of the algorithm. The specific calculation details are in our code. In Schemes C and D, 200 samples are randomly selected from Cluster 2 and Cluster 3, respectively, as additional test sets. The purpose of this experiment is to detect the importance of samples in different clusters for phenotype prediction.

The calculation results are presented in **Figure 6B**. The phenotypic prediction accuracies of Scheme A, B, C, and D are 0.760, 0.661, 0.754, and 0.751, respectively. Their standard deviations are 0.029, 0.033, 0.023, and 0.017 respectively. Undoubtedly, the phenotypic prediction accuracy in Scheme A is the highest. The calculation results of Schemes C and D are close. Scheme B is the worst in terms of both prediction accuracy and prediction stability. If Cluster 1 is reduced by 200 samples, its sample size will only be 51. PNNGS cannot adequately train on Cluster 1 samples. Therefore, the phenotype prediction accuracy in Scheme B drops significantly. Samples in small clusters are more important for phenotype prediction.

**Figure 7:**
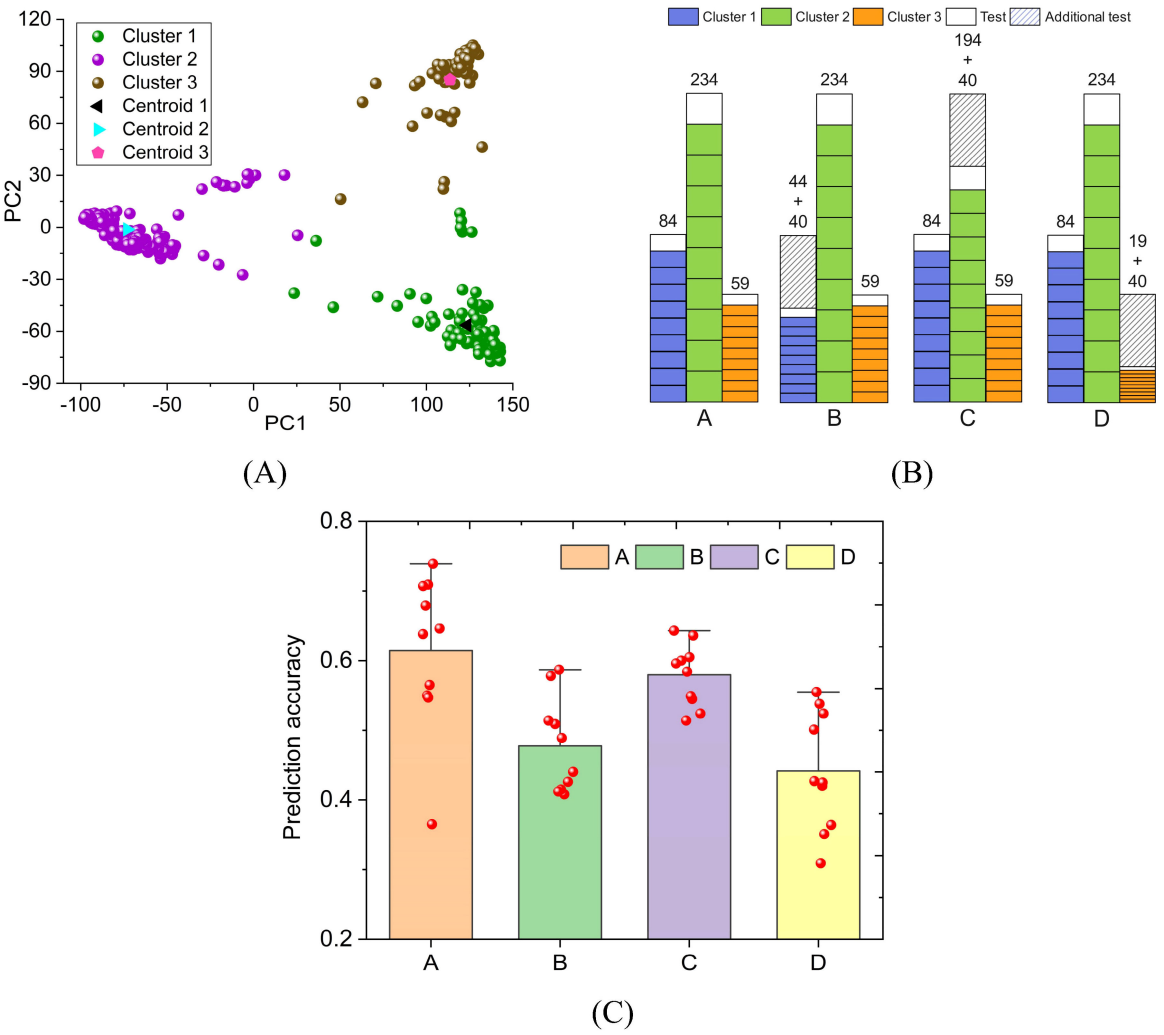
The impact of sample reduction on rice phenotype prediction accuracy. The prediction target is rice FLL. **(A)** PCA and K-means clustering; **(B)** four schemes to divide the FLL training set and test set; **(C)** FLL prediction results of four schemes.

To verify the universality of the above conclusions, we performed similar calculations on rice FLL. The rice genomic data was reduced to two dimensions based on PCA. Rice samples were divided into three clusters by K-means (**Figure 7A**). The number of samples in Clusters 1, 2, and 3 are 84, 234, and 59, respectively (**Supplementary Table S4**). Their centroid coordinates are (123.6, -56.5), (-73.0, -1.2), and (113.6, 85.2), respectively. The four schemes in **Figure 7B** are used for FLL prediction. Due to the small total sample size, 40 samples were selected as an additional test set. In Schemes A, B, C, and D, the prediction accuracy of FLL is 0.614, 0.478, 0.580, and 0.441, respectively (**Figure 7C**). When the sample size of Cluster 3 is reduced by 40, the FLL prediction accuracy decreases significantly (= 0.614-0.441). When the same situation occurs in Cluster 2, the FLL prediction accuracy is only slightly reduced (= 0.614-0.580). The decrease in prediction accuracy is negatively related to the cluster sample size. The FLL standard deviation in Scheme A is 0.106. The standard deviation of FLL is significantly larger than that of GL because the sample size of rice (= 377) is much smaller than that of wheat (= 2000).

In summary, different varieties of plants can be divided into clusters through PCA. Sample sizes can vary widely between clusters. Therefore, our gene files are unbalanced data. Stratified sampling can improve the stability and accuracy of phenotypic prediction. The sample size of small clusters is crucial for phenotypic prediction. If the phenotypic prediction accuracy does not meet the application requirements, increasing the sample size of small clusters is a very effective method. Meanwhile, it can also improve prediction stability.

Compared with the existing GS models, PNNGS shows significant advantages. However, to maximize the prediction accuracy, we recommend training PNNGS in the following way. PNNGS requires a grid search to obtain the optimal number of parallelism. Stratified sampling can improve both the prediction stability and accuracy of PNNGS. We must perform PCA and clustering on the genomic data for stratified sampling. In addition, the more clusters there are, the better the PNNGS prediction is. If the prediction accuracy of PNNGS still cannot meet the application requirements, we need to collect more small cluster samples. The prediction accuracy of PNNGS increases with the increase of phenotypic heritability. We ideally apply PNNGS to phenotypes with high heritability. Current GS models cannot achieve high prediction accuracy for phenotypes with low heritability. Through the above steps, compared with the existing GS model, the prediction accuracy of PNNGS is improved by 0.031, and the prediction standard deviation is reduced by 25%.

## 5 Conclusion

Previous deep learning for GS is serial. Our study introduces parallel structure into GS for the first time. The convolution kernel size of each branch is different. At the same time, residual connections are also added to each branch. Since the Pearson correlation coefficient cannot be a loss function, we train PNGS through four L*p* functions. Through grid search, the optimal parallel numbers for rice, sunflower, wheat, and maize are 4, 6, 4, and 3, respectively. In 24 phenotypic prediction cases of rice, sunflower, wheat, and maize, PNNGS outperformed RRBLUP, RF, SVR, and DNNGP, which shows that PNNGS is highly robust. Compared with DNNGP, the average phenotype prediction accuracy of PNNGS increased by 0.031. From the perspective of NRMSE, PNNGS ranked first in all phenotype predictions. It makes sense for GS to introduce a parallel structure. Random sampling makes phenotypic predictions unstable. Through PCA and K-means, plants can be divided into different clusters. The standard deviations of PL are 0.032, 0.029, and 0.024 through random sampling, 3-cluster stratified sampling, and 100-cluster stratified sampling, respectively. The prediction stability of PNNGS with stratified sampling is significantly improved. PNNGS is trained to predict GL after reducing 200 training samples in each cluster. When reducing samples in small clusters, the prediction accuracy of GL drops significantly. As the number of samples in large clusters decreases, the prediction accuracy of GL decreases slightly. A similar phenomenon occurs with rice. The small cluster sample size is critical for phenotypic prediction. We should collect more plants located in small clusters.

If the attention mechanism is added, the prediction accuracy of PNNGS is expected to be further improved. Meanwhile, the artificially set parameters in PNNGS should be reduced as much as possible. PNNGS is a deep integration of biological technology and information technology in the seed industry. It can breed new varieties of plants and animals faster, better, and more efficiently. With the advancement of deep learning architecture and the increase of plant gene/phenotype data, GS is increasingly showing its superiority.

## Data Availability

The original contributions presented in the study are included in the article/**Supplementary Material**. Further inquiries can be directed to the corresponding author.
